# Prospective, multi-site evaluation of the Cepheid Xpert Xpress CoV-2 *plus* test on nasal and nasopharyngeal swabs

**DOI:** 10.1128/jcm.01219-24

**Published:** 2024-11-06

**Authors:** Alexander L. Greninger, Allan Larcena, Amrish Patel, Brian Webster, Christina Ulen, Dallas F. Green, Dana King, Deepesh Rubin Patel, Erin McElvania, Glenn Harnett, Imad Jandali, Jane Gibson, Jennifer Killion, Jibran Atwi, Kelly Bergmann, Lance Slade, Mary Allen Staat, Matthew Faron, Megan Washington, Rahul Patel, Rajasekaran Annamalai, Ronald Ackerman, William P. Stewart, Yuliet Mora Amador, Deepa Rao, Xiaohong Liu, Aarthi Raman

**Affiliations:** 1 Department of Laboratory Medicine and Pathology, University of Washington, Seattle, Washington, USA; 2 Vaccine and Infectious Disease Division, Fred Hutchinson Cancer Research Center, Seattle, Washington, USA; 3 Slidell Internal Medicine Clinic, Slidell, Louisiana, USA; 4 Innovo Research - Piedmont Healthcare, Troutman, North Carolina, USA; 5 Innovo Research - Wilmington Health, Wilmington, North Carolina, USA; 6 Advanced Pediatrics, Vienna, Virginia, USA; 7 AHF - The Kinder Medical Group, Miami, Florida, USA; 8 University of Iowa Hospitals and Clinics, Iowa City, Iowa, USA; 9 Patient Plus Urgent Care - Southdowns, Baton Rouge, Louisiana, USA; 10 Department of Pathology and Laboratory Medicine, Endeavor Health, Evanston, Illinois, USA; 11 Department of Pathology, University of Chicago, Pritzker School of Medicine, Chicago, Illinois, USA; 12 No Resistance Consulting Group, Birmingham, Alabama, USA; 13 ASCLEPES Research Centers, Brooksville, Florida, USA; 14 UCF Student Health Services, Orlando, Florida, USA; 15 McFarland Clinic - PC, Ames, Iowa, USA; 16 MedPharmics LLC, Lafayette, Louisiana, USA; 17 Children’s of Minnesota, Minneapolis, Minnesota, USA; 18 Meridian Clinical Research LLC, Macon, Georgia, USA; 19 Cincinnati Children’s Hospital and Medical Center, Cincinnati, Ohio, USA; 20 Froedtert & the Medical College of Wisconsin Hospitals and Health Partners, Milwaukee, Wisconsin, USA; 21 TrustCare I-55, Jackson, Mississippi, USA; 22 Meridian Clinical Research LLC, Rockville, Maryland, USA; 23 PCP for Life-Tidwell, Houston, Texas, USA; 24 Comprehensive Clinical Research LLC, West Palm Beach, Florida, USA; 25 Ford, Simpson, Lively & Rice Pediatrics PLLC, Winston-Salem, North Carolina, USA; 26 Santa Clara Family Clinic, Houston, Texas, USA; 27 Cepheid, Sunnyvale, California, USA; Wadsworth Center - NYSDOH, Albany, New York, USA

**Keywords:** SARS-CoV-2, point-of-care, Cepheid, clinical study, rapid, COVID-19, Xpert Xpress

## Abstract

Severe acute respiratory syndrome coronavirus 2 (SARS-CoV-2) continues its largely aseasonal spread with millions of cases per year. Highly sensitive, point-of-care testing is critical for rapid detection of coronavirus disease 2019 (COVID-19) cases and initiation of antiviral therapy to avert adverse health outcomes and reduce onward transmission of the virus. While hundreds of COVID-19 diagnostics received emergency use authorization from the FDA during the pandemic, significantly fewer have navigated the course to FDA clearance or approval. Here, we determined the clinical performance of the Cepheid Xpert Xpress CoV-2 *plus* for detection of SARS-CoV-2 in 3,750 anterior nasal swab (NS) specimens and nasopharyngeal swab (NPS) from 32 sites in comparison to the FDA-authorized BioFire Respiratory Panel 2.1. Three-quarters of specimens collected were tested on the Xpert Xpress CoV-2 *plus* in the point-of-care setting. Overall positive percent agreement (PPA) was 98.1% (95% CI: 96.7%–98.9%) and negative percent agreement (NPA) was 98.3% (97.7%–98.7%). Performance of the Xpert Xpress CoV-2 *plus* was slightly improved in NS compared to NPS specimens, with PPA of 99.3% versus 97.0% (Fisher’s exact test, *P* = 0.06) and NPA of 98.3% versus 98.2% (*P* = 0.89), respectively. Assay PPA was similar between untrained and trained users (98.7% vs 97.3%, *P* = 0.75), while NPA was slightly improved for untrained users (99.0% vs 97.6%, *P* = 0.0003). This study showed that Cepheid Xpert Xpress COV-2 *plus* is highly sensitive and specific/has high PPA and NPA for detection of SARS-CoV-2 from both NS and NPS specimens.

**IMPORTANCE:**

Severe acute respiratory syndrome coronavirus 2 (SARS-CoV-2) continues to cause millions of infections and tens of thousands of deaths per year in the United States. While the FDA authorized hundreds of SARS-CoV-2 tests during the public health emergency, significantly fewer have made the transition to being cleared or approved. There continues to be a need for FDA-authorized point-of-care SARS-CoV-2 testing that can be performed by untrained users. We conducted a large prospective study of the Cepheid Xpert Xpress CoV-2 *plus* test for detection of SARS-CoV-2 in both nasal and nasopharyngeal swabs by trained and untrained users. The assay demonstrated excellent clinical performance characteristics and, as a result of this study, was cleared by the FDA.

## INTRODUCTION

The United States Food and Drug Administration (FDA) granted emergency use authorization to Cepheid’s Xpert Xpress SARS-CoV-2 (first generation) test in March 2020. This sample-to-answer, real-time reverse transcription PCR (RT-PCR) test targeted the nucleocapsid (N2 target) and envelope (E) genes of severe acute respiratory syndrome coronavirus 2 (SARS-CoV-2). Early evaluations of the Xpert Xpress SARS-CoV-2 demonstrated excellent performance characteristics including comparably higher sensitivity than other testing platforms (1–5). Highly sensitive, rapid detection of SARS-CoV-2 is important in increasing efficacy of antiviral therapy and reduction in spread (6).

In March 2021, the FDA reported point mutation(s) in the N2 target of SARS-CoV-2 which caused the first-generation test to detect only the E target in variant viruses (7–9). This led to “Presumptive Positive” callout if only E target was detected and a “Positive” callout if both targets were detected. Thereafter, Cepheid modified Xpress SARS-CoV-2 (first generation) test to develop Xpert Xpress CoV-2 *plus*. Key differences in the Xpert Xpress CoV-2 *plus* test include design changes in the oligo sequences of the primers and probes, differences in the thermal cycling conditions, and the addition of a third gene target (RNA-dependent RNA polymerase, RdRp) in addition to the E and N2 targets (10). The RdRp target was also implemented in the Cepheid Xpert Xpress CoV-2/Flu/RSV *plus* multiplex real-time RT-PCR test. Cepheid performs periodic checks to detect impact of variants on the two tests, and neither test has been affected by variants to date. Few studies have evaluated the test performance characteristics of the Xpert Xpress CoV-2 *plus* (11). Here, we describe the results of a multi-center evaluation of the Xpert Xpress CoV-2 *plus* on prospectively collected, fresh, and frozen nasopharyngeal and anterior nasal swabs.

## MATERIALS AND METHODS

### Specimen collection

This was a multi-site, observational, method comparison study involving prospective fresh and frozen nasopharyngeal swab (NPS) or anterior nasal swab (NS) specimens collected from unique individuals showing signs and symptoms of respiratory infection, including cough, congestion, fatigue, fever with chills, headache, myalgia, nasal discharge, wheezing, new loss of taste and/or smell, nausea, vomiting, diarrhea, or shortness of breath. The specimens were collected at a single point in time when the participants presented themselves at urgent care, clinical outpatient treatment centers, or healthcare organizations providing ambulatory care. Prospectively collected fresh and frozen specimens were enrolled and tested from January 2022 to July 2022 at 32 sites across the United States. One NPS or NS specimen was collected in Copan Universal Transport Medium or Viral Transport Medium from each eligible participant. Specimen collection was conducted according to CDC recommendations for SARS-CoV-2, whereby collection of either a single NPS specimen (i.e., collection from one nostril) was completed using a flocked swab or of anterior nares specimen was completed by sampling both nostrils using a flocked swab. A randomized scheme was used to determine which specimen type was collected from a given participant. Specimens stored at −70°C with ≤1 freeze/thaw cycle and >1.1 mL of specimen volume were enrolled in the study. Consecutive specimens were enrolled during the collection date range and are representative of an all-comer study design.

### Investigational device

The Xpert Xpress CoV-2 *plus* (Cepheid, Sunnyvale, CA, USA) is an automated *in vitro* diagnostic test for the qualitative detection of SARS-CoV-2 RNA. In the clinical study, the performance of Xpert Xpress CoV-2 *plus* was compared with BioFire Respiratory Panel (RP) 2.1 (BioFire Diagnostics, LLC, Salt Lake City, UT, USA) in the detection of SARS-CoV-2 analyte (12). Xpert Xpress CoV-2 *plus* is intended for use with NPS and NS specimens while BioFire RP 2.1 is intended for use with NPS specimens collected from individuals suspected of respiratory tract infections.

The Xpert Xpress CoV-2 *plus* test is performed on the GeneXpert Instrument and Xpress Systems to detect the presence of SARS-CoV-2 RNA in approximately 30 minutes. The GeneXpert Instrument and Xpress Systems automate and integrate sample purification, nucleic acid amplification, and detection of target sequences. The systems require the use of single-use disposable cartridges that hold the PCR reagents and host the PCR process. Non-determinate test results included results of “Invalid,” “Error,” or “No Result” with GeneXpert Instrument Systems and “No Result – Repeat test” and “Instrument Error” with GeneXpert Xpress System. BioFire RP 2.1 is performed on the BioFire FilmArray 2.0 or BioFire FilmArray Torch Systems which automates specimen processing, including cell lysis, nucleic acid capture, amplification, and target detection (12, 13). Discrepancy testing was performed using the Hologic Panther Fusion SARS-CoV-2 test (1, 2). BioFire RP 2.1 targets the spike and membrane genes for detection of SARS-CoV-2, while the Hologic Panther Fusion SARS-CoV-2 test uses two conserved targets within Orf1ab (1, 12).

### Test procedures

Xpert Xpress CoV-2 *plus* testing was performed by trained users on the GeneXpert Instrument System or by untrained users on the GeneXpert Xpress System. In this study, testing occurred in a laboratory setting (*n* = 965 specimens) or in a point-of-care test setting (*n* = 2,785 specimens). All laboratory testing was performed at the University of Washington Virology Lab by trained users on the GeneXpert Instrument System on specimens received from outside sites. Point-of-care testing sites were defined as both near-patient testing (*n* = 891 specimens) and Clinical Laboratory Improvement Amendments (CLIA)-waived testing (*n* = 1,894 specimens). Point-of-care testing was performed in urgent care, clinical outpatient treatment centers, and healthcare organizations providing ambulatory care by a health professional. Near-patient testing was performed on the GeneXpert Instrument for NPS and NS specimens by trained operators, who received instruction on performing the Xpert test and were allowed to practice until they felt comfortable with the test; however, no external competency assessment was performed. CLIA-waived testing was performed on the Xpress System for both NPS and NS specimens with untrained operators who had access to the instructions for use and the one-page pictorial quick reference instructions.

Fresh and frozen specimens were tested per the manufacturer’s Instructions for Use. Frozen specimens were shipped on dry ice and stored at ≤ −70°C. While it was recommended that frozen specimens be tested, including any required repeat testing, on the same day as thawing, operators were instructed to complete all testing of frozen specimens, including any required repeat testing, no later than 48 hours of thawing. After thawing, frozen specimens were stored at 2°C–8°C until testing.

### Performance evaluation

Given that BioFire RP 2.1 does not have an NS claim, a limit of detection comparison study between NPS and NS was conducted to demonstrate adequate comparability between the NPS and NS specimens for use in the clinical study (Table S1). The analytical sensitivity was compared between NS and NPS specimen types for BioFire RP 2.1 using the SARS-CoV-2 isolate USA-WA1/2020 (ATCC, USA) spiked into negative clinical swab matrices at 125, 750, 1,500, and 50,000 copies/mL based on the claimed limit of detection of the BioFire RP 2.1 for NPS specimens (500 copies/mL).

The positive percent agreement (PPA) and negative percent agreement (NPA) for the Xpert Xpress CoV-2 *plus* test in NPS and NS specimens were determined relative to BioFire RP 2.1. Statistical comparisons were performed using Fisher’s exact tests in R v.4.3.0.

## RESULTS

### Sample accountability

**Fig 1:**
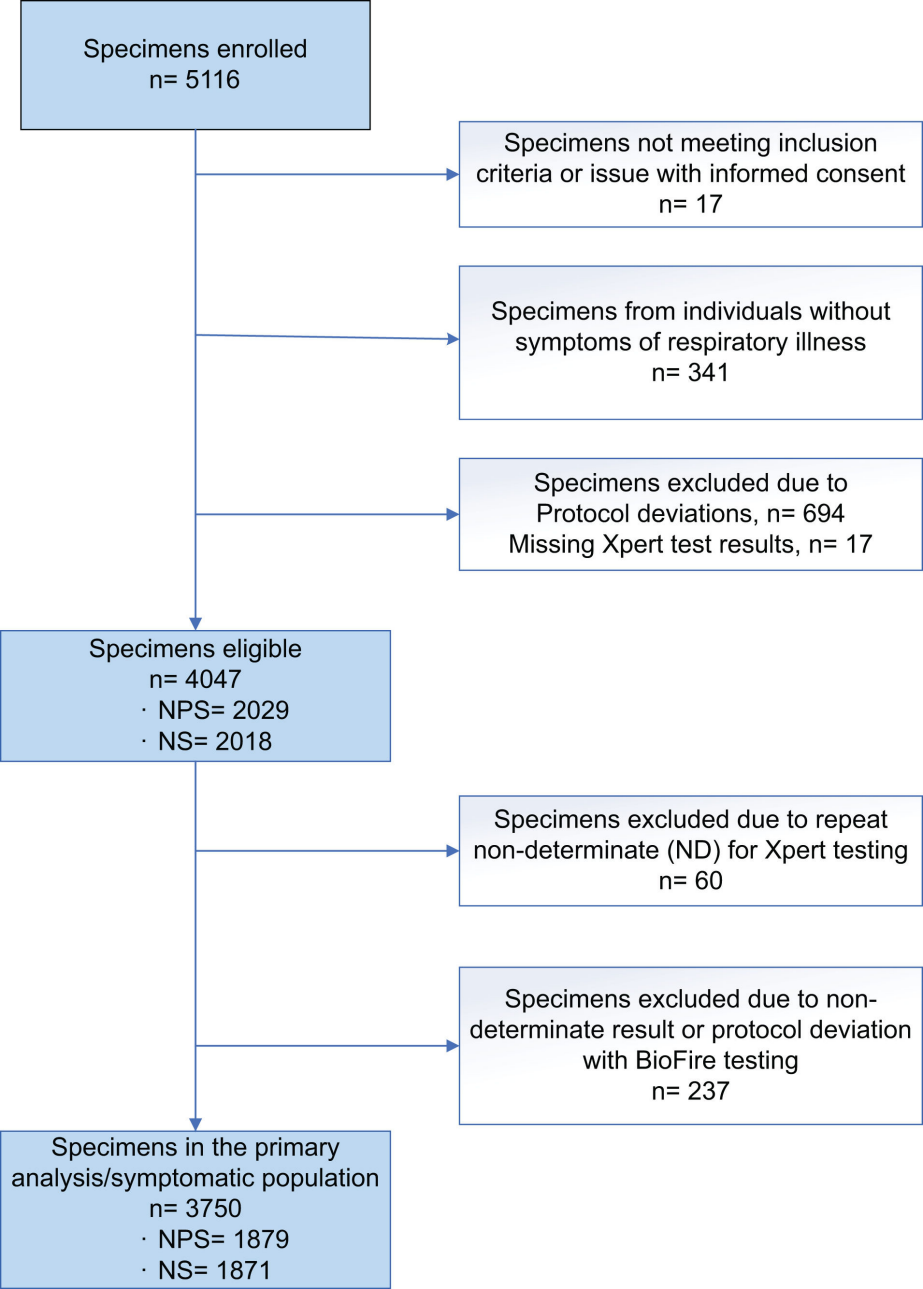
Specimen accountability in the clinical study. CONSORT-like diagram of specimens enrolled and included in the study.

A total of 5,116 NPS and NS specimens were enrolled into this study (Fig. 1). Of these, 17 specimens were found to be ineligible after enrollment and 694 specimens were excluded due to protocol deviations involving procedural deviation with specimen collection, shipping deviation including receiving specimens after stability expiration date, or procedural deviation Xpert testing, wherein the specimen testing was not completed as instructed per protocol. An additional 17 specimens were excluded due to Xpert test and/or BioFire RP 2.1 test results that were not available from testing sites/labs. A total of 341 specimens collected from individuals with no symptoms of respiratory viral illness were excluded from the primary performance analysis (performance in this subset is presented in Table S2). A total of 60 specimens were excluded due to non-determinate testing on Xpert platforms, and an additional 237 specimens were excluded due to non-determinate testing or protocol deviations related to BioFire RP 2.1 testing. A total of 3,750 specimens (1,869 NPS and 1,871 NS) from individuals symptomatic of respiratory infection were included in the performance assessment.

### Demographics

Overall, specimens were collected from slightly more female than male subjects (60.7% and 39.3%, respectively). The study participants were from the following age groups: ≤5 years (1.8%), 6 to 21 years (21%), 22 to 59 years (63.5%), and ≥60 years (13.6%) (Table 1). The preponderance (98.6%) of prospectively collected specimens were tested fresh (Table 1). Due to logistical challenges, a total of 55 (1.4%) of prospectively collected clinical specimens had to be frozen and shipped to a central laboratory site where they were tested on GeneXpert Instrument and the comparator tests.

**TABLE 1:** Demographics of symptomatic individuals, specimen types, and test operators

	Symptomatic of respiratory infection		
	NPS (*N* = 2,029)	NS (*N* = 2,018)	Overall (*N* = 4,047)
Gender			
Female	1,194 (58.8%)	1,262 (62.5%)	2,456 (60.7%)
Male	835 (41.2%)	756 (37.5%)	1,591 (39.3%)
Age group (years)			
≤5	9 (0.4%)	65 (3.2%)	74 (1.8%)
6–21	431 (21.2%)	419 (20.8%)	850 (21.0%)
22–59	1,319 (65.0%)	1,252 (62.0%)	2,571 (63.5%)
≥60	270 (13.3%)	282 (14.0%)	552 (13.6%)
Specimen testing			
Fresh	2,001 (98.6%)	1,991 (98.7%)	3,992 (98.6%)
Frozen	28 (1.4%)	27 (1.3%)	55 (1.4%)
Users			
Trained	1,068 (52.6%)	1,045 (51.8%)	2,113 (52.2%)
Untrained	961 (47.4%)	973 (48.2%)	1,934 (47.8%)
Vaccine status			
Vaccinated	1,435 (70.7%)	1,444 (71.6%)	2,879 (71.1%)
Not vaccinated	572 (28.2%)	553 (27.4%)	1,125 (27.8%)
Unknown	22 (1.1%)	21 (1.0%)	43 (1.1%)

### Performance of Xpert Xpress CoV-2 *plus* for SARS-CoV-2 analyte

The overall results of Xpert Xpress CoV-2 *plus* testing relative to BioFire RP 2.1 for the SARS-CoV-2 analyte detection in symptomatic individuals are presented in Table 2. Of the 3,750 specimens tested, the overall PPA and NPA of the Xpert Xpress CoV-2 *plus* test in NPS and NS specimens compared to BioFire RP 2.1 was 98.1% (574/585) and 98.3% (3,110/3,165), respectively. Performance of the Xpert Xpress CoV-2 *plus* was slightly improved in NS compared to NPS specimens, with PPA of 99.3% versus 97.0% (Fisher’s exact test, *P* = 0.06) and NPA of 98.3% versus 98.2% (*P* = 0.89), respectively. The assay had slightly higher PPA in vaccinated and elderly individuals, while NPA was slightly higher for unvaccinated and younger individuals (Table S3). The positivity rate computed per site and stratified by specimen type and test settings shows equivalent positivity detected between NPS and NS specimens (Table S4).

**TABLE 2:** Performance of Xpert Xpress CoV-2 *plus* in symptomatic individuals

		BioFire RP 2.1								
		Overall			NPS			NS		
		Positive	Negative	Total	Positive	Negative	Total	Positive	Negative	Total
Xpert Xpress CoV-2 *plus*										
Positive		574	55	629	292	28	320	282	27	309
Negative		11	3,110	3,121	9	1,550	1,559	2	1,560	1,562
Total		585	3,165	3,750	301	1,578	1,879	284	1,587	1,871
PPA		98.1% (95% CI: 96.7%–98.9%)			97.0% (95% CI: 94.4%–98.4%)			99.3% (95% CI: 97.5%–99.8%)		
NPA		98.3% (95% CI: 97.7%–98.7%)			98.2% (95% CI: 97.4%–98.8%)			98.3% (95% CI: 97.5%–98.8%)		

### Discrepancy investigation

**Fig 2:**
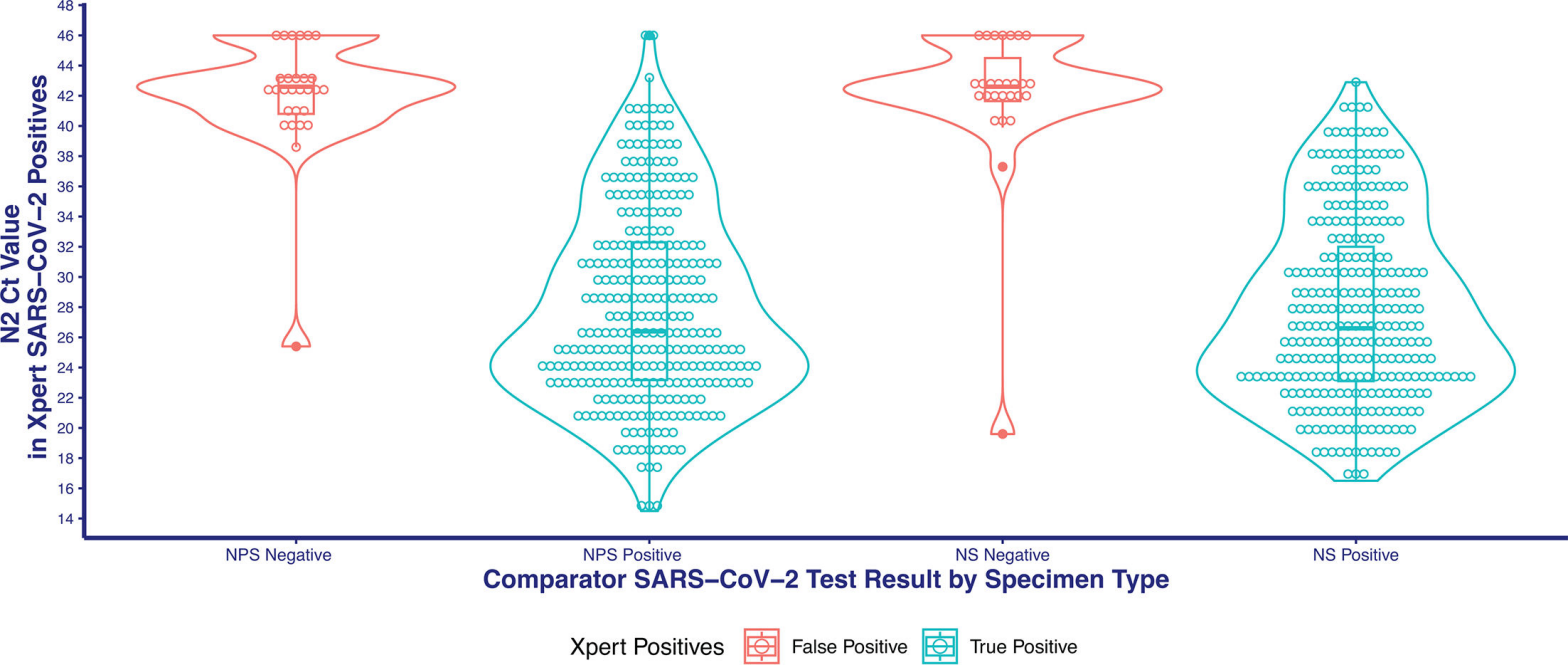
Violin plot of the Ct distribution of Xpert Xpress CoV-2 *plus* test results for NPS and NS specimens compared to the qualitative results of BioFire RP 2.1 comparator. N2 gene target Ct values are depicted for positive Xpert Xpress CoV-2 *plus* test results based on BioFire RP 2.1 comparator results. Specimens testing positive on the Xpert Xpress CoV-2 *plus* test based on E or RdRp gene target detection but negative for the N2 target are plotted above the assay’s cutoff for the N2 target. Filled-in circles are considered outliers based on >1.5× interquartile range.

A total of 55 (28 NPS and 27 NS) specimens yielded false positive results by Xpert Xpress Cov-2 *plus* compared to BioFire RP 2.1 (Table S5). False positive NPS and NS tests were associated with specimens with lower viral load compared to the overall testing population (Fig. 2). Discrepant analysis was performed using the Hologic Panther Fusion SARS-CoV-2 test. On discrepancy investigation, 16 (8 NPS and 8 NS) specimens yielded a positive result, 37 (19 NPS and 18 NS) specimens yielded a negative result, and 2 (1 NPS and 1 NS) specimens yielded an invalid result. Of the 28 NPS specimens that yielded false positive results, 27 specimens were tested fresh, and 1 specimen was tested frozen. Of the 27 NS specimens that yielded false positive results by Xpert Xpress CoV-2 *plus*, 24 specimens were tested fresh and 3 specimens were tested frozen.

There were 11 (9 NPS and 2 NS) specimens that yielded a false negative result by Xpert Xpress CoV-2 *plus*. Upon discrepant testing, 9 (7 NPS and 2 NS) specimens yielded a negative result, 1 NPS specimen yielded a positive result, and 1 NPS specimen yielded an invalid result when tested with Hologic Panther Fusion SARS-CoV-2. Of the 11 specimens, 9 NPS and 1 NS specimens were tested fresh, and 1 NS specimens was tested frozen.

### Xpert Xpress CoV-2 *plus* performance stratified by trained and untrained users

The performance of Xpert Xpress CoV-2 *plus* with trained users using GeneXpert Instrument Systems and with untrained users using GeneXpert Xpress System is presented in Table 3. Performance of Xpert Xpress CoV-2 was evaluated in 1,967 specimens tested by trained users and 1,783 specimens tested by untrained users. The PPA of the assay in the detection of SARS-CoV-2 analyte was similar between trained and untrained users, at 97.8% and 98.6%, respectively (Fisher’s exact test, *P* = 0.75). The NPA of the Xpert Xpress CoV-2 *plus* was significantly higher for untrained compared to trained users (99.1% vs 97.4%, *P* = 0.0003).

**TABLE 3:** Xpert Xpress CoV-2 *plus* performance by users

Analyte	User	PPA (95% CI)	NPA (95% CI)
SARS-CoV-2	Untrained	98.6% 215/218 (96.0%–99.5%)	99.1% 1,551/1,565 (98.5%–99.5%)
	Trained	97.8% 359/367 (95.8%–98.9%)	97.4% 1,559/1,600 (96.5%–98.1%)

### Xpert Xpress CoV-2 *plus* performance stratified by test settings

The performance of Xpert Xpress CoV-2 plus stratified in laboratory and point-of-care test settings is presented in Table 4. The Xpert Xpress CoV-2 plus test performed similarly in both settings, with a PPA of 96.9% and 98.4% (*P* = 0.40) and NPA of 97.6% and 98.5% (*P* = 0.09) in laboratory and point-of-care settings, respectively.

**TABLE 4:** Xpert Xpress CoV-2 *plus* performance by test settings

Analyte	User	PPA (95% CI)	NPA (95% CI)
SARS-CoV-2	Laboratory	96.9% 94/97 (91.3%–98.9%)	97.6% 847/868 (96.3%–98.4%)
	Point-of-care testing	98.4% 480/488 (96.8%–99.2%)	98.5% 2,263/2,297 (97.9%–98.9%)

## DISCUSSION

Here, we describe a large prospective multi-center evaluation of Xpert Xpress CoV-2 *plus* test using almost 4,000 NPS and NS specimens from symptomatic individuals. Overall, the Xpert Xpress CoV-2 *plus* had high positive percent agreement (>97%) and high negative percent agreement (>98%) compared to the BioFire RP 2.1, the first molecular test approved by the FDA for qualitative detection of SARS-CoV-2.

Based on the results of this evaluation, the FDA cleared the Xpert Xpress CoV-2 *plus* in October 2023 as a class II device for the qualitative detection of SARS-CoV-2 RNA in nasopharyngeal and anterior nasal swab specimens collected from individuals with signs and symptoms of respiratory tract infection. The Xpert Xpress CoV-2 *plus* became the 13th molecular assay out of only 18 total to be cleared by the FDA for qualitative detection of SARS-CoV-2 more than 1 year after the end of the public health emergency, compared to 274 SARS-CoV-2 molecular tests given emergency use authorization during the pandemic. Of the 18 cleared tests, the Xpert Xpress CoV-2 *plus* is one of seven tests that only assay for SARS-CoV-2 and do not include other respiratory pathogen targets. As SARS-CoV-2 continues to spread in a near-aseasonal fashion and remains a significant cause of preventable hospitalization and mortality (14), offering rapid, point-of-care testing for SARS-CoV-2 alone may be helpful for insurance reimbursement outside of the winter months. Conversely, labs may prefer to validate and offer only one multiplex test that at least covers SARS-CoV-2, influenza A/B viruses, and respiratory syncytial virus, in addition to other respiratory pathogens, given workflow, training, and quality control considerations (15). The continued zoonotic threat of H5N1 influenza virus in the USA and other countries may also drive year-round respiratory virus multiplex testing.

Overall, Xpert Xpress CoV-2 *plus* performed well when using nasopharyngeal or nasal swabs and whether users were trained or untrained and regardless of laboratory or point-of-care testing location. Of note, assay performance of the Xpert Xpress CoV-2 *plus* was slightly better with NS compared to NPS and when testing was performed by untrained users compared to trained users. Positivity rates by testing site for NS versus NPS specimens in both CLIA-waived and near-patient testing were similar (Table S4). Taken together, these data suggest that the current widespread use of nasal swabs in combination with a sensitive molecular test achieves adequate performance characteristics for SARS-CoV-2 testing, with the limitation that NS and NPS specimens were not taken from the same individual in this study. Evaluation of assay performance with untrained operators is required for CLIA waiver decisions and, during the pandemic, was commonly reported for antigen tests (16). For the Xpert Xpress MVP test for vaginitis, untrained users also had a statistically stronger negative percent agreement for the *Candida glabrata/krusei* target with simultaneous improved positive percent agreement, highlighting the ease of use for untrained users of the Xpress Instrument system (17). Similarly, the assay performed slightly better in the point-of-care setting compared to the laboratory setting, though this difference was not statistically significant. Though statistically significant, the absolute difference in performance between trained and untrained users in these contexts is small and thus essentially equivalent in overall test performance.

Limitations of the study include the use of a qualitative comparator in the BioFire RP 2.1 test, which was largely dictated based on regulatory considerations. This prevented full comparison of Ct values between the two platforms; however, this is a secondary consideration given the qualitative authorization of both tests. Individuals under 5 years of age were also somewhat underrepresented in the study population. Approximately 8.7% (55/629) of positive Xpert Xpress CoV-2 *plus* assay results were not detected by the BioFire RP 2.1, though 43.6% (24/55) of these results occurred in January–March 2022 when SARS-CoV-2 prevalence was high at testing sites. However, these limitations are minor compared to the strengths of the study, including the large number of specimens examined and strong clinical performance characteristics of the Xpert Xpress CoV-2 *plus* assay across specimen type, testing site, and user setting. In summary, the Xpert Xpress CoV-2 *plus* assay is a highly reliable assay for the qualitative molecular detection of SARS-CoV-2 in nasopharyngeal and anterior nasal swab specimens collected from individuals with signs and symptoms of respiratory tract infection.

## SUPPLEMENTAL MATERIAL

Supplemental tables jcm.01219-24-s0001.xlsx Tables S1 to S5.

ASM does not own the copyrights to Supplemental Material that may be linked to, or accessed through, an article. The authors have granted ASM a non-exclusive, world-wide license to publish the Supplemental Material files. Please contact the corresponding author directly for reuse.
